# Shared Genetic Basis of Autoimmune Diseases and Follicular Lymphoma by Mendelian Randomization and Multi-Omics

**DOI:** 10.3390/genes17091031

**Published:** 2026-08-28

**Authors:** Chendong Jiang, Zhan Sun, Zhanyan Gao, Jie Wang, Jun Liang, Yang Feng

**Affiliations:** 1 Department of Laboratory Medicine, The Third Affiliated Hospital of Zhengzhou University, Zhengzhou 450052, China; jianyanjiang@zzu.edu.cn; 2 Department of Dermatology, Huashan Hospital, Fudan University, Shanghai 200040, China; 24111220061@m.fudan.edu.cn (Z.S.); 21211220028@m.fudan.edu.cn (Z.G.); wangjiedemuma@163.com (J.W.); 3 Department of Dermatology, Massachusetts General Hospital, Harvard Medical School, Boston, MA 02114, USA; 4 Cutaneous Biology Research Center, Department of Dermatology, Massachusetts General Hospital, Boston, MA 02114, USA

**Keywords:** mendelian randomization, pleiotropy, follicular lymphoma, autoimmune diseases, immunogenetics

## Abstract

**Background/Objectives**: Epidemiological studies link autoimmune diseases (AIDs) to follicular lymphoma (FL) risk, but their shared genetic architecture and causal mechanisms remain unclear. **Methods**: A two-sample Mendelian randomization (MR) analysis was employed to assess causal relationships between 15 AIDs and FL. Pleiotropic loci were identified through the Pleiotropy Analysis under Composite Null (PLACO). Bayesian colocalization analysis, functional mapping, and Multi-marker Analysis of GenoMic Annotation were applied to fine-map shared genetic variants and identify their target genes. Summary data-based MR was used with multitissue expression quantitative trait locus data to infer causal effects of gene expression. HyPrColoc analysis was applied to decipher shared genetic regulation of immune cell phenotypes. **Results**: MR revealed that rheumatoid arthritis increased FL risk (OR_IVW_ = 1.55, nominal *p* = 7.16 × 10^−5^, FDR-corrected *p* = 1.07 × 10^−3^), whereas composite autoimmune disease reduced FL risk (OR_IVW_ = 0.70, nominal *p* = 4.61 × 10^−5^, FDR-corrected *p* = 6.92 × 10^−4^). Hypothyroidism showed only a nominally suggestive protective trend (OR_IVW_ = 0.88, nominal *p* = 0.015), which did not survive Benjamini–Hochberg multiple-testing correction (FDR-corrected *p* = 0.075). Fifty-five pleiotropic loci shared between FL and AIDs were identified, among which key loci such as **1p36.32**, **6p21.32**, **17p13.1***,* and **11q23.3** exhibited strong colocalization evidence. Core pleiotropic genes (e.g., *TNFRSF14*, *MMEL1*, *CXCR5,* and *RNASET2*) were prioritized, which implicated pathways related to MHC class II antigen presentation, interferon signaling, and T cell activation. HyPrColoc analysis demonstrated that these loci colocalized with the expression of immune receptors, including BAFF-R on B cells and HVEM (*TNFRSF14*) on naïve CD8^+^ T cells. **Conclusions**: Our study identifies divergent causal effects of selected AIDs on FL risk and demonstrates localized pleiotropy at key loci, providing novel insights into shared immunogenetic mechanisms.

## 1. Introduction

Follicular lymphoma (FL), a germinal center B cell malignancy, arises within a chronically dysregulated immune microenvironment. Epidemiological evidence firmly links autoimmune diseases (AIDs) to increased FL risk, albeit with disease-specific variation: Sjögren’s syndrome (SS) confers the highest risk (Standardized Incidence Ratio, SIR = 4.9), followed by systemic lupus erythematosus (SLE; SIR = 4.4) and rheumatoid arthritis (RA; SIR = 2.0) [1,2]. This association is particularly pronounced in younger patients, with an SIR of 2.21 for FL in AIDs patients diagnosed with non-Hodgkin lymphoma (NHL) before age 60 [1] and is corroborated by an independent study in a Chinese cohort (odds ratio, OR ≈ 1.6) [3]. Critically, RA-comorbid FL portends a poor prognosis, evidenced by low complete response (33%) and 5-year event-free survival (48–56%) rates [4]. These collective observations underscore the clinical imperative to dissect the shared mechanisms underlying FL-AID comorbidity for guiding early intervention and refining therapeutic strategies.

The mechanisms linking AIDs and FL may involve chronic inflammation-driven disruption of the immune microenvironment and/or the role of immunosuppressive agents (such as TNF antagonists) in reactivating latent Epstein–Barr virus (EBV) infection [3,5]. Molecularly, shared genetic susceptibility is implicated, notably within the human leukocyte antigen (HLA) class II region. Genome-wide association studies (GWASs) have pinpointed amino acid position 13 of HLA-DRB1 as a common risk locus for both FL and AIDs, potentially amplifying comorbidity risk [6]. However, the following key questions persist: Do different AIDs confer FL risk through distinct biological pathways? What shared genetic or epigenetic loci exist beyond the HLA region? Addressing these questions is critical for elucidating common pathogenesis and informing targeted immunomodulatory strategies. Globally, the prevalence of AIDs has steadily increased over the past three decades, partially attributable to rising environmental pollution and altered dietary patterns [7]. Against this backdrop, unravelling the shared genetic basis between AIDs and FL gains further clinical relevance. Consequently, an integrated genetic analytics framework—spanning causal inference, genetic correlation, pleiotropy analysis, functional annotation, and colocalization—was applied to systematically delineate the shared genetic architecture between AIDs and FL.

## 2. Materials and Methods

### 2.1. Study Design and Data Sources

To minimize population stratification, the analyses were based on publicly available GWAS summary statistics from individuals of European ancestry. The primary data sources were the FinnGen Consortium (Release 12) and the GWAS Catalog, with detailed sample sizes and accession codes listed in Table S1. Phenotypes from FinnGen included FL, composite autoimmune disease (AD), RA, hypothyroidism (HT), childhood asthma, asthma, Graves’ disease, inflammatory bowel disease, Crohn’s disease, ulcerative colitis, multiple sclerosis, ankylosing spondylitis, autoimmune hyperthyroidism, and psoriasis. The GWAS Catalog data comprised adult-onset asthma (AOA, GCST007799), primary biliary cirrhosis (PBC, GCST90061440), SLE (GCST003156), and vitiligo (GCST004785). AD is a FinnGen Release 12 meta-phenotype aggregating autoimmune sub-phenotypes; RA and HT, used as separate MR exposures, are components of this trait. Full definitions and sub-phenotype details are provided in the footnote of Supplementary Table S1. Additional datasets were incorporated for integrative analysis: (i) Immunophenotypes: Summary data for 731 immune cell traits from the GWAS Catalog (accession range: GCST90001391–GCST90002121). (ii) Expression QTLs (eQTLs): eQTL data from the GTEx v8 project (54 tissues, https://gtexportal.org/, accessed on 10 March 2025) and the eQTLGen Consortium (whole blood, 2022 release, https://www.eqtlgen.org/, accessed on 11 March 2025 ). Linkage Disequilibrium (LD) Reference: European subset from the 1000 Genomes Project Phase 3. A standardized quality control and harmonization pipeline was applied: variants were aligned to the forward strand and lifted to GRCh37/hg19, ambiguous palindromic SNPs (MAF > 0.42) were excluded, and variants with large allele frequency differences (>0.3) from the reference panel were removed. Of note, several exposure phenotypes and the FL outcome were obtained from FinnGen Release 12, where partial participant overlap across different disease phenotypes cannot be fully excluded. We reused the cross-trait intercept derived from LD score regression (LDSC), which had already been calculated as part of the Pleiotropy Analysis under Composite Null (PLACO) pleiotropy workflow, to quantitatively evaluate potential bias caused by sample overlap and population stratification for our MR analysis. The intercept value was close to 1, suggesting limited bias originating from shared study participants.

### 2.2. Mendelian Randomization (MR) Analysis

A two-sample MR framework assessed causal relationships in three dimensions: from AIDs to FL, from AIDs/FL to immune cell traits, and from AIDs/FL to eQTLs/pQTLs. Instrumental variables (IVs) were selected based on a three-stage protocol: (1) variant-exposure association significance (*p* < 5 × 10^−8^); (2) LD-based clumping (clump_kb = 10,000; clump_r^2^ = 0.001) to ensure independence; and (3) assessment of instrument strength (F-statistic > 10). After allele alignment, primary causal estimates were generated using inverse-variance weighted (IVW) regression. For MR analyses of 15 AID exposures on FL, Benjamini–Hochberg false discovery rate (FDR) correction was applied across all 15 tests, with FDR–corrected *p* < 0.05 as the significance threshold, while nominal raw *p*-values were also reported. Sensitivity analyses included the MR-Egger and weighted median methods. Robustness was evaluated by testing for horizontal pleiotropy (MR-Egger intercept), heterogeneity (Cochran’s Q), and via MR PRESSO outlier correction, single-SNP analysis, leave-one-out testing, and funnel plot inspection.

### 2.3. Pleiotropy Analysis

Loci with pleiotropic effects on FL and AIDs were identified using the PLACO method [8]. For each trait pair, GWAS summary statistics were harmonized by aligning effect alleles, deriving Z-scores, and excluding variants with zero standard error or extreme Z-scores. The covariance matrix of Z-scores was estimated via the var.placo function using independent SNPs (*p* < 1 × 10^−4^). The intercept from cross-trait LDSC [9] was incorporated into the covariance matrix to adjust for potential sample overlap. The PLACO test statistic was computed for each SNP under the null hypothesis of no pleiotropy (H0: β_1_β_2_ = 0). A sensitivity analysis was conducted using the 95% confidence interval of the LDSC intercept.

### 2.4. Functional Annotation and Gene Mapping

Identified risk loci were functionally annotated and mapped to genes using the Functional Mapping and Annotation (FUMA) platform [10]. This involved: (1) Variant Annotation of functional consequences, (2) Pathogenicity Scoring (ANNOVAR, CADD, RegulomeDB; annotation resources internally implemented within the FUMA platform), and (3) Gene Mapping through positional (±10 kb), eQTL (GTEx v8), and chromatin interaction strategies. FUMA was used to annotate the pleiotropic loci identified by PLACO, map lead SNPs to genes, and perform enrichment analyses. Gene-based and gene-set analyses were conducted using Multi-marker Analysis of GenoMic Annotation (MAGMA) v1.10 [11] for independent validation. SNPs were mapped to genes based on physical position (NCBI Build 38, ±10 kb window). Gene-level association statistics were computed by aggregating summary statistics of SNPs within each gene, adjusting for LD structure using the 1000 Genomes European reference panel. Gene Set Enrichment Analysis (GSEA) was performed using gene sets from MSigDB (v2024.1), including: canonical pathways (C2: curated gene sets), Gene Ontology terms (C5: biological processes, molecular functions, cellular components), and transcription factor targets (C3: TFT). Tissue-specific expression enrichment was assessed using GTEx v8 data. All tests were corrected for gene size, GC content, and multiple testing (FDR < 0.05 threshold).

### 2.5. Colocalization Analysis

Bayesian colocalization analysis was performed to determine whether FL and AIDs share causal genetic variants within associated genomic regions [12]. For each locus of interest (±500 kb around a lead SNP with *p* < 5 × 10^−8^), summary statistics were harmonized. The coloc.abf function was then applied to compute the posterior probabilities (PPs) for the five competing hypotheses regarding variant sharing. Loci with a posterior probability for a shared causal variant (PP.H4) ≥ 0.7 were considered strong evidence for colocalization.

### 2.6. Transcriptomic/Proteomic Integration (SMR Analysis)

Summary data-based Mendelian randomization (SMR) [13] was implemented to test for potential causal effects of gene expression on disease risk. This method uses cis-eQTLs as instrumental variables. cis-eQTL summary data were integrated from three immunologically relevant tissues in GTEx v8 [14]: whole blood, spleen, and EBV-transformed lymphocytes. LD information was obtained from the 1000 Genomes Project European panel (Phase 3 v5). The SMR test significance threshold was set at *p* < 0.05 after FDR correction for the number of genes tested per tissue. To distinguish causal association from linkage (i.e., to confirm the eQTL and GWAS signal were driven by the same variant), the heterogeneity in dependent instruments (HEIDI) test was applied; results with a HEIDI test *P*-value < 0.01 were considered to reflect linkage and were excluded. To further mitigate false positives from horizontal pleiotropy, we prioritized genes that were jointly identified by both FUMA (genomic mapping) and MAGMA (gene-based association, FDR < 0.05).

### 2.7. Multi-Trait Colocalization with Immunophenotypes

Multi-trait colocalization analysis using HyPrColoc [15] was employed to identify shared causal variants underlying FL, AIDs, and immunophenotypes. Genomic regions and immune traits were pre-selected based on significant associations from IVW-MR (FDR *p* < 0.05) and PLACO/FUMA results. For each locus, all available variants within the candidate region were harmonized by aligning allele effects, standardizing allele frequency and effect directions across traits. Effect size (β) and standard error (SE) matrices were analyzed with HyPrColoc, applying a regional probability threshold of 0.8. Consistent with software requirements, only loci containing a minimum of 3 high-quality SNPs were included for analysis. We computed posterior probabilities for regional colocalization (PP_regional_), SNP-level driving variant (PP_snp_), and an overall estimate (PP_overall_). Colocalization was considered significant when PP_regional_ ≥ 0.80 and the lead SNP’s PP_snp_ > 0.90. Finally, all significant results were subjected to FDR correction (*p* < 0.05) and annotated with immune cell metadata for biological interpretation.

### 2.8. Statistical and Computational Tools

The analysis integrated multiple computational environments. Core statistical analyses and visualization were conducted in R (v4.4.2) utilizing specialized packages for MR (v0.6.9), MR PRESSO (v1.0), pleiotropy analysis (PLACO (v0.1.1)), colocalization (coloc (v5.2.3) and HyPrColoc (v0.0.2)). Specific genetic analyses employed dedicated command-line tools: LDSC (v1.0.1) for genetic correlation, MAGMA (v1.10) for gene-set analysis, and SMR (v1.4.0) for transcriptome-wide integration. The genomic coordinate liftover utilized UCSC LiftOver tools, and functional annotation was performed via the FUMA web platform (v1.8.0, https://fuma.ctglab.nl, accessed on 12 May 2025). All statistical tests performed in this study were two-tailed. Multiple-testing correction was performed via the Benjamini–Hochberg FDR procedure. All MR, genetic-correlation and colocalization analyses were performed following previously published standard analytical workflows.

## 3. Results

### 3.1. MR Identifies Divergent Causal Effects of AIDs on FL

**Figure 1:**
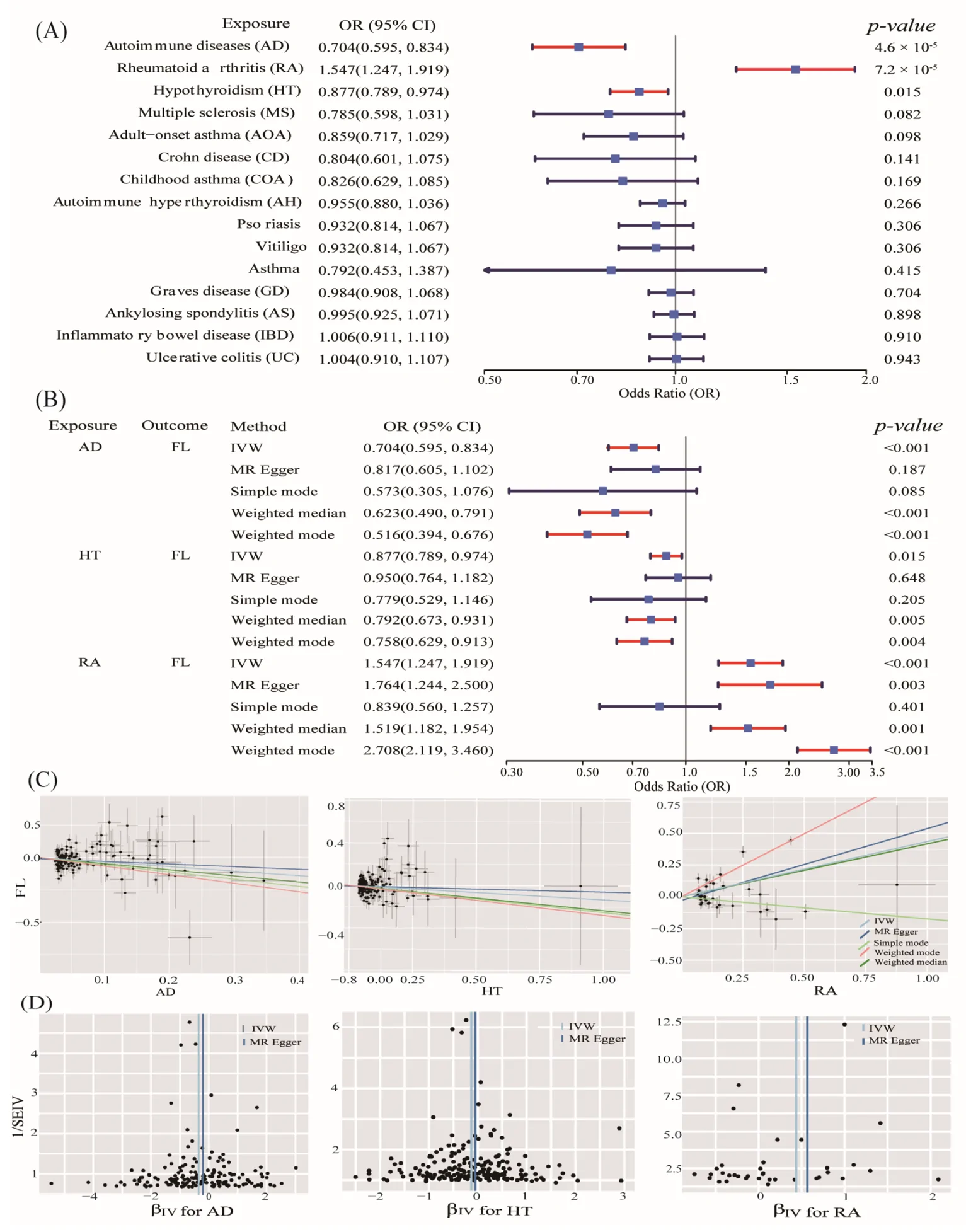
MR analysis of causal relationships between AIDs and FL. (**A**) Forest plot of causal effects of AIDs on FL risk using the IVW method. (**B**) Forest plot of significant AIDs (AD, HT, RA) with FL using multiple MR methods. Statistically significant associations are highlighted in red in panels (**A**,**B**). (**C**) Scatter plots of genetic associations of AD, HT, and RA with FL risk. Black dots represent individual genetic variants (SNPs). Grey lines represent 95% confidence intervals for each variant. Solid lines represent IVW estimates. (**D**) Funnel plots for MR analyses of AD, HT, and RA with FL, assessing pleiotropy and robustness of results. Abbreviations: AD, composite autoimmune disease; HT, hypothyroidism; RA, rheumatoid arthritis; FL, follicular lymphoma; IVW, inverse-variance weighted; OR, odds ratio; CI, confidence interval.

A two-sample MR analysis was used to assess the causal effects of 15 AIDs on FL risk (Figure 1). IVW estimates revealed divergent effects: AD and HT significantly reduced FL risk (AD: OR = 0.70, 95% CI: 0.60–0.83, nominal *p* = 4.61 × 10^−5^, FDR *p* = 6.92 × 10^−4^, whereas RA significantly increased risk (OR = 1.55, 95% CI: 1.25–1.92, nominal *p* = 7.16 × 10^−5^, FDR *p* = 1.07 × 10^−3^). HT showed a nominally suggestive protective trend (OR = 0.88, 95% CI: 0.79–0.97, nominal p = 0.015), which failed Benjamini–Hochberg correction (FDR *P* = 0.075). These causal estimates were robust across sensitivity analyses (weighted median, weighted mode) and showed no significant evidence of horizontal pleiotropy (MR-Egger intercept *P* > 0.05 for all). MS did not show a statistically significant association in the IVW analysis (OR = 0.79, 95% CI: 0.60–1.03, *p* = 0.082), although the weighted median method suggested a potential protective effect (OR = 0.86, *p* = 0.016). No associations reaching FDR significance were observed for the remaining exposures (Table S2).

### 3.2. Identification and Functional Analysis of Shared Pleiotropic Loci BetweenFL and AIDs

**Figure 2:**
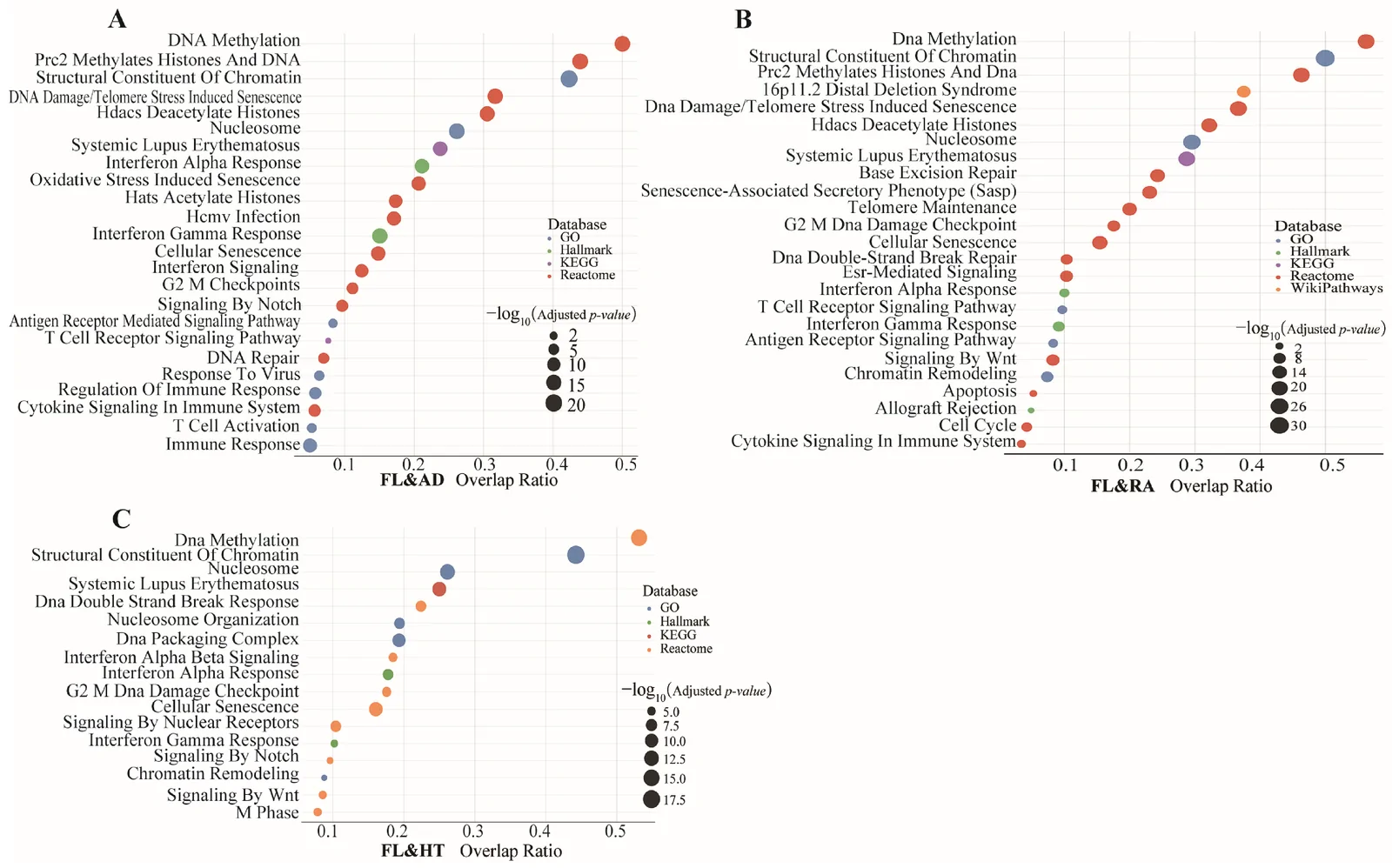
Bubble plot of FUMA gene set enrichment analysis based on PALCO pleiotropy results. (**A**) FL-AD; (**B**) FL-RA; (**C**) FL-HT. X-axis shows gene overlap ratio. Bubble size corresponds to −log10 (adjusted *p*-value), bubble color denotes gene-set database source. Gene sets are sorted in descending order by overlap ratio. Significance threshold: adjusted *p* = 0.05. Panel-specific size breaks were applied to accommodate the broad range of significance values across datasets.

Despite the significant causal effects identified by the MR analysis, LDSC revealed no significant genome-wide genetic correlation between FL and AIDs (Table S3). This intriguing observation suggests that the shared genetic architecture is likely driven by locus-specific pleiotropy rather than a broad polygenic overlap. Based on this hypothesis, PLACO was performed to identify specific loci jointly influencing FL and AD, RA, or HT. The LDSC intercept confirmed minimal bias from sample overlap or population stratification, which ensured the robustness of the pleiotropic analysis. PLACO analysis identified 55 pleiotropic loci shared between FL and AD, RA, or HT (FL&AD: 22; FL&RA: seven; FL&HT: 26; Table S4, Figures S1 and S2). Loci **1p36.32, 6p21.32***,* and **2q33.3** were shared across all three trait pairs (Table S5, Figures S3–S5). As summarized in Table 1, Bayesian colocalization (PP.H4 ≥ 0.7) supported shared causal variants at *17p13.1* for FL&AD (PP.H4 = 0.985), and at **11q23.3** (PP.H4 = 0.917), **3p24.1** (PP.H4 = 0.857), and **16p12.1** (PP.H4 = 0.700) for FL&RA (Figure S6). Colocalization evidence was weaker for FL&HT, with the top locus **1p13.2** (PP.H4 = 0.446) not reaching significance (Table S4). FUMA annotation of 145 lead SNPs revealed predominantly intronic (27.6%) and intergenic (46.9%) variants (Table S6). Cross-trait gene sets identified 814 (FL&AD), 1277 (FL&HT), and 651 (FL&RA) genes, primarily comprising protein-coding genes, pseudogenes, and non-coding RNAs. GSEA revealed that pleiotropic loci are implicated in pathways including nucleosome assembly and histone modification, DNA damage repair and cellular senescence, and interferon-mediated immune responses. Notably, significant enrichment was observed across all three diseases for nucleosome-associated gene sets (e.g., GO cellular component: nucleosome), DNA methylation pathways (e.g., Reactome: DNA methylation), and interferon response pathways (e.g., Hallmark: Interferon gamma response) (Figure 2; Table S7). Functional enrichment results from Figure 2 highlight key shared biological modules, including interferon-mediated immune responses, lymphocyte activation, and chromatin-remodeling pathways, pointing to immune dysregulation and epigenetic perturbation as core shared pathogenic features linking FL and AIDs.

### 3.3. MAGMA Functional and Tissue-Specific Enrichment of Shared FL–Autoimmune Susceptibility Loci

**Figure 3:**
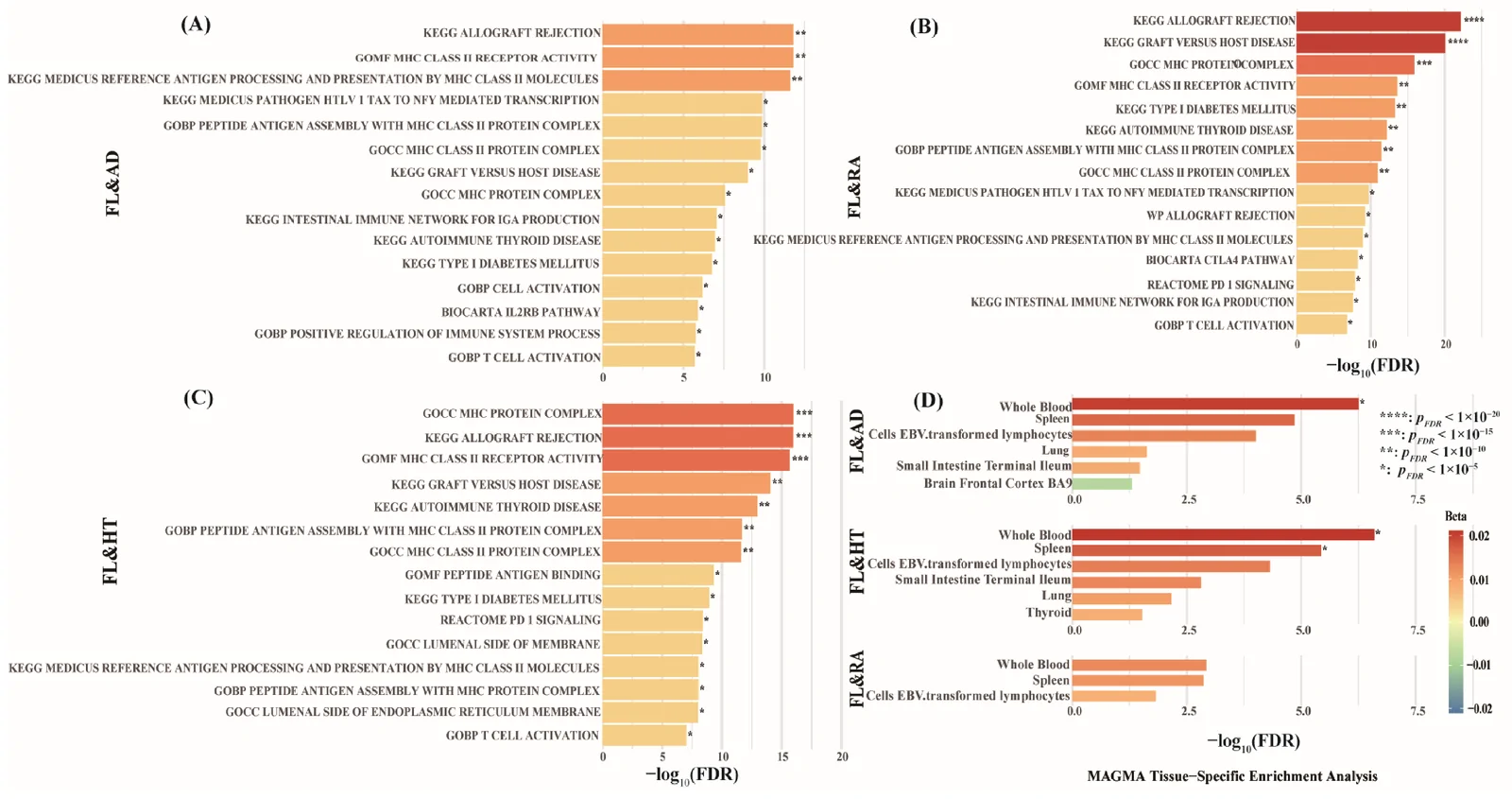
Bar plots of MAGMA gene set enrichment (**A**–**C**) and tissue specificity (**D**) analyses based on PALCO pleiotropy results. Note: FL, follicular lymphoma; AD, composite autoimmune disease; RA, rheumatoid arthritis; HT, hypothyroidism.

Complementing FUMA, MAGMA gene-set enrichment analysis identified significant shared genetic susceptibility between FL and AD, HT, or RA, which converged on immune pathways. Universally enriched pathways across all trait pairs included MHC class II antigen presentation (e.g., GO Molecular Function: MHC class II receptor activity; KEGG: Antigen processing and presentation) and alloimmunity (KEGG: Allograft rejection; KEGG: Graft-versus-host disease). Disease-specific enrichments were observed for Autoimmune thyroid disease (KEGG) in FL&HT and Genes associated with RA development (WikiPathways) in FL&RA. Additional enriched pathways involved T cell activation (GO Biological Process: T-cell activation), PD-1 signaling (Reactome: PD-1 signaling), and cytokine pathways—including Cytokine signaling in immune system (Reactome) and IL-6-JAK-STAT3 signaling (Hallmark) in FL&AD, and JAK-STAT signaling pathway (KEGG) in FL&RA—highlighting roles for MHC class II presentation, T cell immunity, and cytokine networks in FL–autoimmune genetic pleiotropy (Figure 3A–C; Tables S8 and S9). MAGMA tissue-specificity analysis demonstrated a significant enrichment of shared genetic effects in immune-related tissues. Whole blood showed the strongest associations (FL&AD: *p* = 5.61 × 10^−7^; FL&HT: *p* = 2.54 × 10^−7^), followed by the spleen and EBV-transformed lymphocytes. FL&HT also exhibited specific enrichment in thyroid tissue (*p* = 0.0296). These findings underscore the importance of hematopoietic and lymphoid tissues—particularly whole blood and spleen—in the shared genetic architecture of FL and AIDs, supporting their prioritization in subsequent tissue-specific SMR analyses (Figure 3D; Table S10).

### 3.4. Integrated Multi-Omics Evidence Identifies High-Priority Pleiotropic Genes and Their Functional Roles

**Figure 4:**
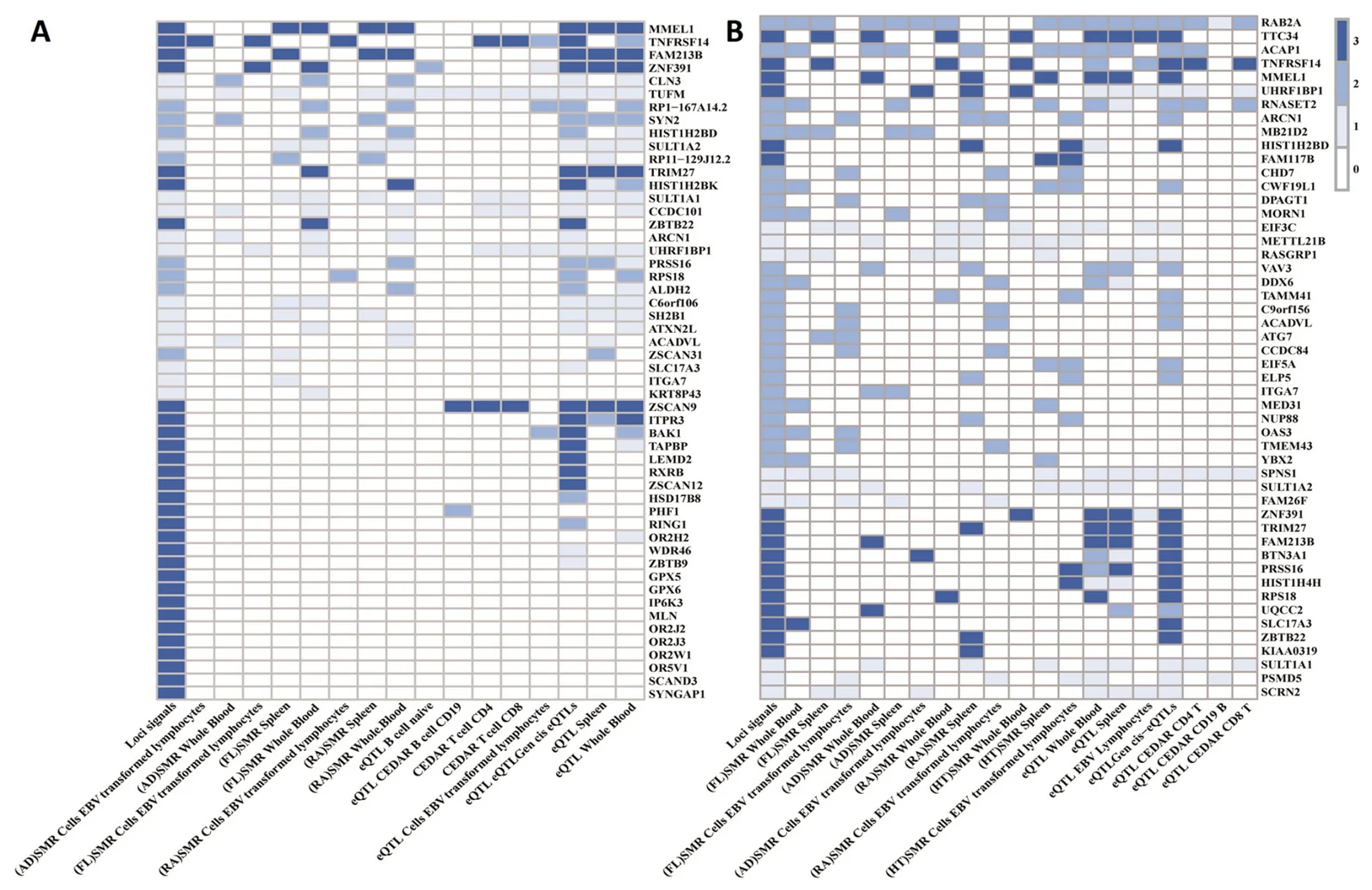
Heatmap of key pleiotropic genes based on MAGMA (A) and FUMA (B). Note: The signals represent the number of gene hits across different trait pairs. Loci signal indicates the frequency of being mapped across different trait pairs. eQTL, expression quantitative trait locus; SMR, summary-data-based Mendelian randomization; FL, follicular lymphoma; AD, composite autoimmune disease; RA, rheumatoid arthritis; HT, hypothyroidism.

To prioritize candidate genes from the pleiotropic loci identified by PLACO, gene mapping results from both FUMA and MAGMA were integrated, which identified 222 overlapping genes, 30 of which were shared across all three trait pairs (Table S11). Based on convergent evidence from cross-trait associations, multi-tissue SMR, and multi-source eQTL validation, several high-confidence candidate hub genes were prioritized, including *TNFRSF14*, *MMEL1*, *TUFM*, *SULT1A1*, *CLN3*, and *SYN6* (Figure 4A; Table S12). To further refine the functionally supported set of pleiotropic genes, stringent filtering was applied based on FUMA’s integrated mapping evidence (positional, eQTL, and chromatin interaction data) combined with cross-tissue SMR significance, which yielded 260 high-confidence pleiotropic genes (Figure 4B; Table S13). The comprehensive profiles of these genes are detailed in Table S14. Among 194 genes mapped to ≥2 trait pairs, five core genes exhibited consistent evidence across all three diseases and multiple tissues: *TNFRSF14*, *MMEL1*, *UHRF1BP1*, *TTC34,* and *HIST1H2BD*. Four others (*RAB2A*, *ACAP1*, *ARCN1,* and *MB21D2*) were strongly supported for two diseases. Tissue-specific associations (e.g., *RNASET2* for AD/RA/HT in spleen, CHD7 across traits in whole blood, and autophagy-related ATG7 for AD in EBV lymphocytes/whole blood) highlight spleen, whole blood, and EBV-transformed B cells as key regulatory microenvironments.

**Figure 5:**
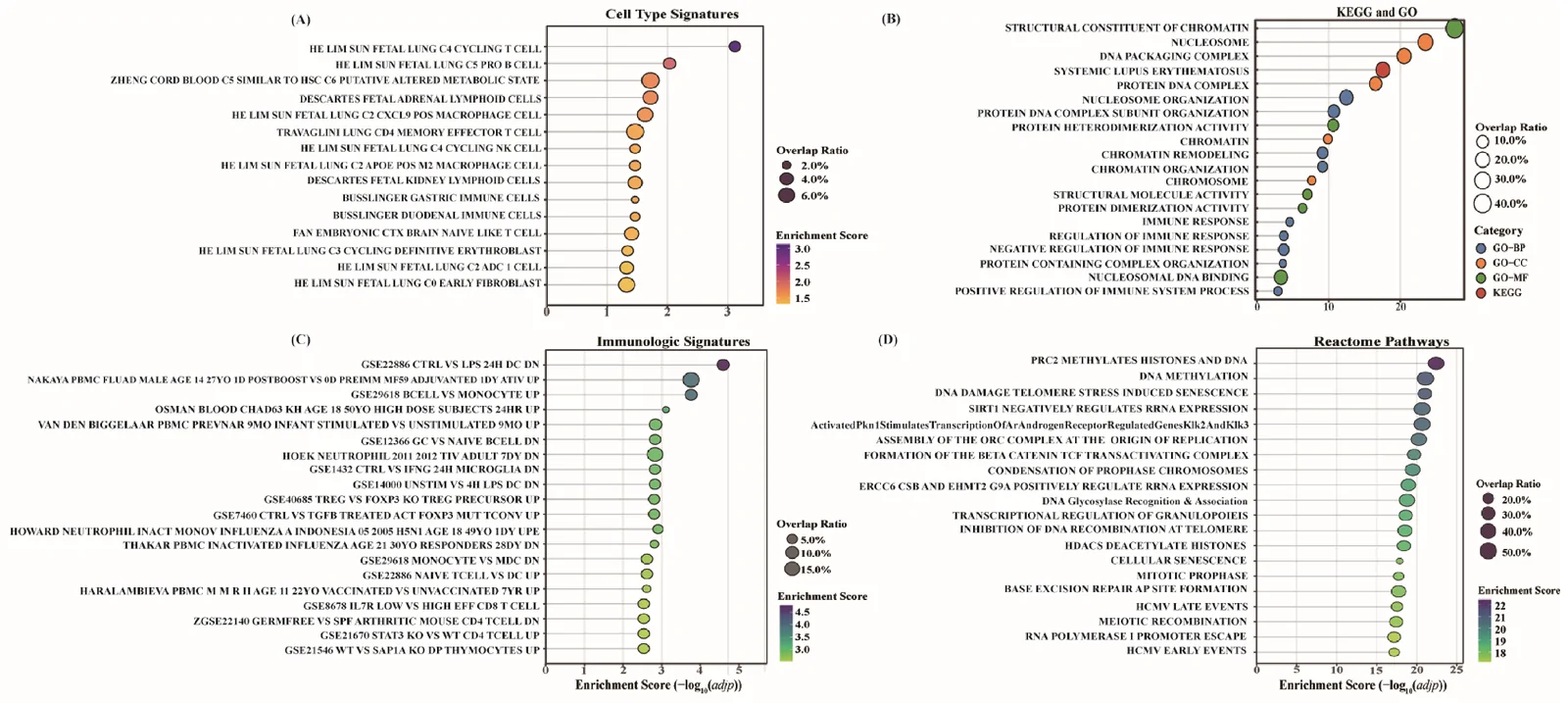
Bubble plot of gene set enrichment analysis for pleiotropic genes. (**A**) Cell type signatures; (**B**) KEGG and GO signaling pathways; (**C**) immunologic signatures; (**D**) Reactome pathways.

Given the immune-related nature of both FL and AIDs, we assessed the expression profiles of these pleiotropic genes across the major immune tissues (Figures S7 and S8). Cross-tissue analysis of the 21 genes detected in all three tissues revealed that 75% (16/21), including the antiviral genes *OAS1* and *OAS2* and the apoptosis regulator *BCL2*, showed significantly elevated expression in EBV-transformed lymphocytes. Conversely, 95% (20/21) exhibited highly concordant expression between the spleen and whole blood, indicating systemic coordination. Histone genes (e.g., *HIST1H2BJ*) maintained consistently low expression across all tissues, suggesting stable epigenetic regulation (Tables S15 and S16). Notably, *ACAP1* and *RNASET2* demonstrated consistently high expression across all three tissues, while *CXCR5* showed elevated expression specifically in the spleen and blood. Functional enrichment analysis revealed that pleiotropic genes were significantly associated with chromatin assembly (structural constituent: *p* < 10^−28^; organizational processes: *p* < 10^−12^), the systemic lupus erythematosus pathway (*p* = 2.73 × 10^−18^), and immune regulation (immune response: *p* < 0.05) (Figure 5C). These genes were primarily enriched in fetal immune cells, particularly fetal lung cycling T cells (*p* = 7.59 × 10^−4^) and pro-B cells (*p* = 9.19 × 10^−3^) (Figure 5A), where they mediated disruption of immune tolerance (LPS-tolerized dendritic cell downregulation: *p* = 1.67 × 10^−5^) and aberrant activation (vaccine-responsive genes: *P* < 0.0013), alongside B cell differentiation blockade (germinal center vs. naïve B cell inhibition: *p* = 0.0011) and immune cell dysfunction (*p* < 0.0025) (Figure 5B). Reactome pathway analysis further implicated these genes in epigenetic reprogramming (PRC2-mediated methylation: *p* < 10^−22^), telomere stress-induced senescence (*p* = 9.39 × 10^−22^), and dysregulated DNA repair (*p* < 10^−18^), collectively contributing to genomic instability (Figure 5D).

### 3.5. Multi-Trait Colocalization Analysis Uncovers Immune Cell Phenotype Regulation by Shared Loci

Significant immune cell traits identified by MR analysis of FL/AD/RA/HT against 731 phenotypes were further analyzed using HyPrColoc to probe shared genetic regulation (Table S17). This approach identified significant genetic colocalization between FL and AD/HT/RA at four genomic loci (**17p13.1**, **11q23.3**, **12q24.12**, and **1p36.32**), implicating 137 distinct immune cell phenotypes (Table S18). The **17p13.1** locus was predominantly associated with FL&AD, exerting broad regulatory effects on regulatory T cells, monocytes, dendritic cells (DCs), and B cells. The **11q23.3** locus primarily governs FL&RA associations, strongly targeting B cells (particularly B cell-activating factor receptor (BAFF-R) expression), monocytes, and DC phenotypes. The **12q24.12** locus drives shared FL&AD/HT phenotypes, specifically modulating the CD14^+^ CD16^+^ monocyte subset. Strikingly, both the **17p13.1** (FL&AD) and **11q23.3** (FL&RA) loci demonstrated robust genetic colocalization with BAFF-R expression levels across multiple B cell subsets (regional probability [Reg. Prob] = 0.8324 for **17p13.1**/0.9848 for **11q23.3**; Overall PP = 0.8255 for **17p13.1**/0.9163 for **11q23.3**). These subsets included CD20^−^ B cells, CD24^+^ CD27^+^ B cells, IgD^−^ CD38^bright^ plasma blasts, IgD^+^ CD38^−^ unswitched memory B cells, memory B cells, and transitional B cells. Furthermore, significant genetic colocalization was observed between several loci and herpesvirus entry mediator (*HVEM*/*TNFRSF14*) expression levels on naïve CD8^+^ T cells. For FL&AD, strong colocalization evidence was detected at **17p13.1** (Reg. Prob = 0.8325, Overall PP = 0.8258; lead SNP rs314258) and **1p36.32** (Reg. Prob = 0.9931, Overall PP = 0.8505). For FL&RA, in addition to sharing the signal at **1p36.32**, loci at **11q23.3** (Reg. Prob = 0.9848, Overall PP = 0.9163) and **16p12.1** (Reg. Prob = 0.8017, Overall PP = 0.5182) were also identified.

## 4. Discussion

In this study, the integration of multidimensional genetic approaches enabled the first systematic elucidation of the complex causal relationships and shared biological mechanisms between AIDs and FL. The MR analysis indicated that composite AD was associated with a significant reduction in FL risk, whereas RA was associated with a significant increase in FL risk. HT only showed a nominally suggestive protective trend, which did not survive Benjamini–Hochberg multiple-testing correction and thus lacked convincing causal evidence. These causal associations were validated using multiple robust MR approaches, and sensitivity analyses indicated the absence of significant pleiotropic interference, thereby confirming the reliability of these results. Notably, despite the robust causal effects observed for AD and RA from MR, LDSC detected no significant genetic correlations between FL and AD/HT/RA. This paradoxical observation suggests that the FL-AID associations are likely driven by locally shared pleiotropic loci rather than genome-wide genetic overlap. This pattern, where disease-relevant pleiotropic signals reside in discrete loci without detectable genome-wide genetic correlation, has been described in autoimmune cancer genetic studies. A study of multiple sclerosis and common cancers identified multiple shared pleiotropic loci including lymphoma-associated variants, despite an absence of significant genome-wide genetic correlation [16]. It supports the notion that comorbidity can be shaped by locus-specific pleiotropy rather than broad polygenic sharing. To elucidate this mechanism, PLACO analysis was performed, which identified 55 significant pleiotropic loci; loci **1p36.32**, **6p21.32**, and **2q33.3** were found to jointly influence all three disease pairs. Using an integrated approach comprising Bayesian colocalization, FUMA gene annotation, and MAGMA analysis, high-priority candidate pleiotropic hub genes (e.g., *TNFRSF14* and *MMEL1*) were prioritized. Their functional importance was further validated by cross-tissue SMR and multi-source eQTL data. Crucially, HyPrColoc analysis demonstrated that these loci specifically regulate BAFF-R and HVEM (*TNFRSF14*) expression across multiple immune cell subsets, providing mechanistic insights at the cellular phenotype level for FL-AID comorbidity mechanisms. Collectively, these identified pleiotropic loci exert pivotal immunomodulatory functions in B cell survival and T cell-mediated immune homeostasis, which partly explains the shared genetic pathogenesis of AIDs and FL.

The genetically predicted increase in FL risk from RA (OR= 1.55) is directionally consistent with prior observational studies (SIR ≈ 2.0), with the attenuated effect size likely reflecting MR’s robustness against confounding by treatment or surveillance bias [1,17,18]. This difference may reflect the ability of MR to minimize confounding by treatment and surveillance biases. In contrast, the protective effects of AD and HT observed in MR (OR ≈ 0.70–0.88) conflict with neutral or elevated risks reported in epidemiological studies [1,2,3,4]. This divergence may arise from both methodological and biological factors. First, MR captures lifelong genetic susceptibility, including subclinical immune states, whereas cohort studies typically assess short-term risks after clinical diagnosis [19]. Subclinical or autoantibody-positive states may confer protective immune activity that restrains premalignant B cell proliferation. Consistent with this, our analyses revealed a shared enrichment in immune activation pathways—including interferon signaling, T cell activation, cytokine responses, and cell cycle regulation—across AIDs. Moreover, elevated *TNFRSF14* expression may enhance CD8^+^ T cell-mediated tumor surveillance. Second, observational studies may be influenced by immunosenescence, cumulative inflammation [20,21], and immunosuppressive treatments [22,23] in clinically diagnosed patients, which could mask the protective genetic effects. For instance, immunosuppression-related reactivation of latent EBV infection is one established mechanism contributing to an increased NHL risk [24].

Integrated multi-omics analyses established a causal link between FL and AIDs and underscored the central role of the *TNFRSF14*/*HVEM* axis at **1p36.32**. Colocalization and cross-tissue SMR analyses supported the association of this locus with FL, AD, and RA, particularly in EBV-transformed lymphocytes. HVEM functions as both a receptor—activating NF-κB to promote survival—and a ligand—engaging BTLA on T cells to deliver inhibitory signals [25,26]. Genetic analyses further revealed colocalization between this locus and HVEM expression on naïve CD8^+^ T cells. In FL, loss of HVEM facilitates immune evasion; high-frequency mutations or deletions alleviate T cell suppression, promoting malignant transformation [25,26,27]. This defect originates in in situ follicular neoplasia and is often retained during clonal evolution [28]. Conversely, in AID-susceptible individuals, intact HVEM-BTLA signaling maintains immune homeostasis by restraining T cell hyperactivation, a mechanism validated in BTLA-deficient models and reflected in balanced differentiation of naïve CD8^+^ T cells [5,28,29,30]. Thus, *TNFRSF14*/HVEM serves as a unified molecular basis for FL-AID comorbidity: its functional deficiency drives FL immune escape, whereas its intact activity safeguards immune equilibrium in AIDs. Furthermore, at the **17p13.1** locus—which shows strong colocalization evidence with AD (PP.H4 > 0.98)—significant colocalization with HVEM expression on naïve CD8^+^ T cells was also observed, suggesting that this region may contribute to disease pathogenesis by modulating immune microenvironment phenotypes. The key gene within this region, *TP53*, is an important tumor suppressor whose dysfunction is closely associated with various cancers. Although *TP53* was not directly mapped in this study, its key regulator, *WRAP53*, was successfully identified. *WRAP53* directly modulates *TP53* transcription and expression through an “antisense RNA regulatory” mechanism [31].

The **11q23.3** locus (rs73005502; rs4291686), located near *CXCR5*, has been associated with FL and vitiligo [32,33] and showed strong colocalization evidence as a shared risk locus for RA and lymphoma [34]. This region overlaps with EBV-associated chromosomal aberrations and includes *SIK3*, which helps maintain cytotoxic T cell responses [31]. HyPrColoc analysis delineated two key regulatory axes at this locus: (1) A BAFF-R signaling axis, where the variant rs314258 colocalized with BAFF-R expression across multiple B cell subsets. As a central regulator of B cell survival and proliferation, BAFF-R critically contributes to FL pathogenesis, progression, and treatment resistance, representing a promising therapeutic target [35]. (2) A CCR2 immunomodulatory axis, where rs10892279 colocalized with CCR2 expression on monocytes and myeloid dendritic cells. Intriguingly, elevated CCR2 on monocytes/mDCs is associated with reduced risk of DLBCL and MZL, whereas high CCR2 on granulocytes increases susceptibility to FL and MZL, highlighting its cell context-dependent role [36]. The **16p12.1** locus, previously implicated in SLE and other AIDs [37] and linked to endometrial cancer risk via genes, such as *EIF3C* and *SPNS1* [38], was newly identified here as a shared risk locus for FL and RA. It also showed genetic colocalization with HVEM expression on naïve CD8^+^ T cells.

Tissue-specific analyses highlighted the spatial and functional architecture of the shared genetic basis between FL and AIDs, revealing significant enrichment of genetic effects in whole blood and spleen. This underscores the importance of systemic immunity and secondary lymphoid organs in FL–AD comorbidity and suggests synergistic roles of systemic immune dysregulation and local microenvironmental perturbations. Cross-tissue SMR mapping prioritized spleen-specific genes, such as *RNASET2* and *CXCR5*. *RNASET2* is a lymphoma-associated antigen highly expressed in the spleen and a colocalized plasma protein associated with RA and autoimmune thyroid disease [39,40,41]. *CXCR5* plays important roles in both AIDs (e.g., RA, SSC, SLE) and FL: in the former, it regulates B cell and follicular helper T cell functions involved in disease initiation and progression [42,43,44]; in the latter, it mediates B cell positioning and retention in the germinal center microenvironment [45], reflecting functional consistency across diseases. Observations from EBV-transformed lymphocytes revealed marked upregulation of pleiotropic genes (e.g., *OAS1*/*OAS2*, *BCL2*). These cell line-derived findings generate a hypothesis that latent EBV infection could potentiate genetic risk via aberrant activation of antiviral, anti-apoptotic, and proliferative pathways in B cells, though they do not constitute direct in vivo evidence for EBV acting as a major environmental trigger in patients [46].

Several limitations must be considered. First, although MR suggested opposing effects of AD/HT versus RA on FL risk—potentially explained by differing immune stimulation dynamics—further functional characterization could help dissect the underlying biological mechanisms responsible for these divergent causal patterns, while MR-derived genetic evidence itself supports our causal inference. Second, despite divergent effect directions among AIDs, pleiotropy analyses revealed shared immune pathways (e.g., MHC class II presentation, interferon signaling). Genes such as *TNFRSF14* and *MMEL1* influenced FL risk across autoimmune contexts, but their effects may depend on immune microenvironment status, warranting further investigation. Moreover, although MR reduces confounding, it remains challenging to fully exclude potential influences of medications (e.g., immunosuppressants in RA) on FL risk. Additionally, no significant genetic associations were detected for certain AIDs (e.g., SLE, SS), possibly due to limited power or distinct biological mechanisms; larger studies are needed. Third, several exposure and FL GWAS summary statistics originated from FinnGen, so partial participant overlap cannot be fully excluded. Though cross-trait LDSC intercept and MR sensitivity analyses supported result robustness, future replication using fully independent FL GWAS datasets is still desirable. Furthermore, eQTL observations obtained from EBV-transformed lymphoblastoid cell lines reflect regulatory features of an immortalized in vitro model. These findings cannot directly validate whether latent EBV infection represents a major environmental trigger for the shared genetic associations among FL and AIDs in patients. Finally, as this study primarily used data from European-ancestry populations, caution is required when generalizing the findings to other populations. Including more diverse cohorts will be essential to enhance the generalizability of the results.

## 5. Conclusions

In conclusion, this study systematically elucidated the causal relationships and shared genetic architecture between AIDs and FL through integrated multi-omics analyses. Several cross-disease pleiotropic loci have been identified, including **1p36.32**, **6p21.32**, **17p13.1**, and **11q23.3**. Genes such as *TNFRSF14*, *MMEL1*, *CXCR5*, and *RNASET2* exhibited consistent cross-disease co-expression and regulation across tissues (whole blood, spleen, and EBV-transformed lymphocytes), indicating their central roles in the comorbidity of FL–AIDs. Furthermore, these shared genetic loci showed significant colocalization with key immune phenotypes, including B cells (e.g., BAFF-R), monocytes, and T cells (e.g., HVEM expression on naïve CD8^+^ T cells), highlighting the pivotal involvement of B cell survival and T cell regulatory pathways in the shared etiology of these diseases.

## Figures and Tables

**Table 1:** Shared genetic loci between FL and AIDs.

Trait Pairs	Region	Locus Boundary	Lead SNP	Position	*p*_PLACO_	PP.H4
FL&AD	17p13.1	17:7085965–7240391	rs61759532	17:7240391:C:T	1.48 × 10^−9^	0.985
FL&RA	11q23.3	11:118482565–118746433	rs73005502	11:118697906:A:G	5.02 × 10^−15^	0.917
FL&RA	3p24.1	3:27757018–27800734	rs12497690	3:27795397:A:C	6.97 × 10^−10^	0.857
FL&RA	16p12.1	16:28338039–28920320	rs11074907	16:28613965:A:G	9.94 × 10^−9^	0.700
FL&RA	1p36.32	1:2470671–2721149	rs60733400	1:2516781:A:G	6.18 × 10^−10^	0.217
FL&AD	2q33.3	2:204746767–205022390	rs34556908	2:204782282:C:CT	7.45 × 10^−9^	0.214
FL&RA	2q33.3	2:204690355–204794757	rs10932028	2:204793063:A:C	4.35 × 10^−10^	0.177
FL&HT	2q33.3	2:204746767–205022390	rs34556908	2:204782282:C:CT	6.72 × 10^−10^	0.167
FL&AD	1p36.32	1:2470671–2725952	rs4073285	1:2539796:C:T	9.79 × 10^−14^	0.163
FL&HT	1p36.32	1:2483961–2721149	rs60389769	1:2717470:A:G	1.34 × 10^−9^	0.008
FL&HT	6p21.32	6:33173842–33856598	rs439205	6:33173842:A:G	5.11 × 10^−14^	0.000
FL&AD	6p21.32	6:33173842–34342104	rs186384986	6:33467086:A:T	9.70 × 10^−22^	0.000
FL&RA	6p21.32	6:33172306–33873375	rs62405954	6:33524820:C:T	1.47 × 10^−19^	0.000

Note: This table integrates the shared genetic loci between FL and AIDs (RA, HT, and AD). The upper section shows loci with strong colocalization evidence (PP.H4 ≥ 0.7), whereas the lower section lists loci recurrently identified across all three trait pairs. Genomic coordinates are based on the GRCh37/hg19 assembly. Abbreviations: SNP, single-nucleotide polymorphism; PP.H4, posterior probability for hypothesis H4; lead SNP, the top lead SNP (smallest P-values) in genomic loci; FL, follicular lymphoma; AD, composite autoimmune disease; RA, rheumatoid arthritis; HT, hypothyroidism.

## Data Availability

This study analyzes existing publicly available data (FinnGen Release 12, GWAS Catalog). The sources of all data are explicitly cited in the Section 2 and detailed in Table S1.

## Supplementary Materials

The following supporting information can be downloaded at https://www.mdpi.com/article/10.3390/genes17091031/s1, Figure S1. Manhattan plot of the PLACO results; Figure S2. QQ plots for pleiotropic results performed by PLACO; Figure S3. Key shared genetic loci between FL and AD; Figure S4. Key shared genetic loci between FL and RA; Figure S5. Key shared genetic loci between FL and HT; Figure S6. Regional association plots for loci with significant genetic colocalization between FL and AIDs; Figure S7. Tissue-specific expression patterns of pleiotropic genes; Figure S8. Gene-set enrichment for identified pleiotropic genes. Table S1. Data sources; Table S2. MR analysis and sensitivity analysis; Table S3. Genetic correlation analysis between FL and AIDs; Table S4. Shared pleiotropic loci identified by PLACO; Table S5. Shared pleiotropic loci among different trait pairs; Table S6. Mapped genes of pleiotropic loci identified by PLACO; Table S7. GSEA revealed significant sharing of multiple biological pathways between FL and AD/HT/RA; Table S8. MAGMA genes of pleiotropic loci identified by PLACO; Table S9. MAGMA gene-set analysis; Table S10. MAGMA tissue-specific analysis; Table S11. Concordant gene mapping results from FUMA and MAGMA; Table S12. Candidate hub genes refined from the FUMA and MAGMA gene intersection; Table S13. Pleiotropic genes identified through integrated evidence from SMR, multi-database eQTL, and ciMap; Table S14. Information on pleiotropy genes identified; Table S15. Expression value of pleiotropy genes identified in different tissues from GTEx; Table S16. Tissue-specific enrichment of pleiotropy genes identified in different tissues from GTEx; Table S17. Summary of Mendelian randomization analyses between AID, FL, and immune cell traits; Table S18. Multi-trait colocalization analysis highlighted key role of immune cells (PP > 0.8).

## Abbreviations

The following abbreviations are used in this manuscript.

AIDs	autoimmune diseases
FL	follicular lymphoma
MR	Mendelian randomization
SLE	systemic lupus erythematosus
EBV	Epstein–Barr virus
HLA	human leukocyte antigen
GWASs	genome-wide association studies
AD	composite autoimmune disease
RA	rheumatoid arthritis
HT	hypothyroidism
LD	linkage disequilibrium
IVW	inverse-variance weighted
FDR	false discovery rate
PLACO	pleiotropy analysis under composite null
LDSC	linkage disequilibrium score regression
FUMA	functional mapping and annotation
MAGMA	multi-marker analysis of GenoMic annotation
GSEA	gene set enrichment analysis
PPs	posterior probabilities
SMR	summary data-based Mendelian randomization
SNP	single-nucleotide polymorphism
