# Supplementary material for: Shared Genetic Basis of Autoimmune Diseases and Follicular Lymphoma by Mendelian Randomization and Multi-Omics
Source: Genes (Basel). 2026 Aug 28;17(9):1031. doi: 10.3390/genes17091031 (PMC13606217; doi:10.3390/genes17091031)

Figure S1. Manhattan plot of the PLACO results. Note: Red line represents the significance of 5×10^{-8}. Abbreviations: FL, Follicular lymphoma; AD, Composite autoimmune disease; RA, Rheumatoid arthritis; HT, Hypothyroidism.

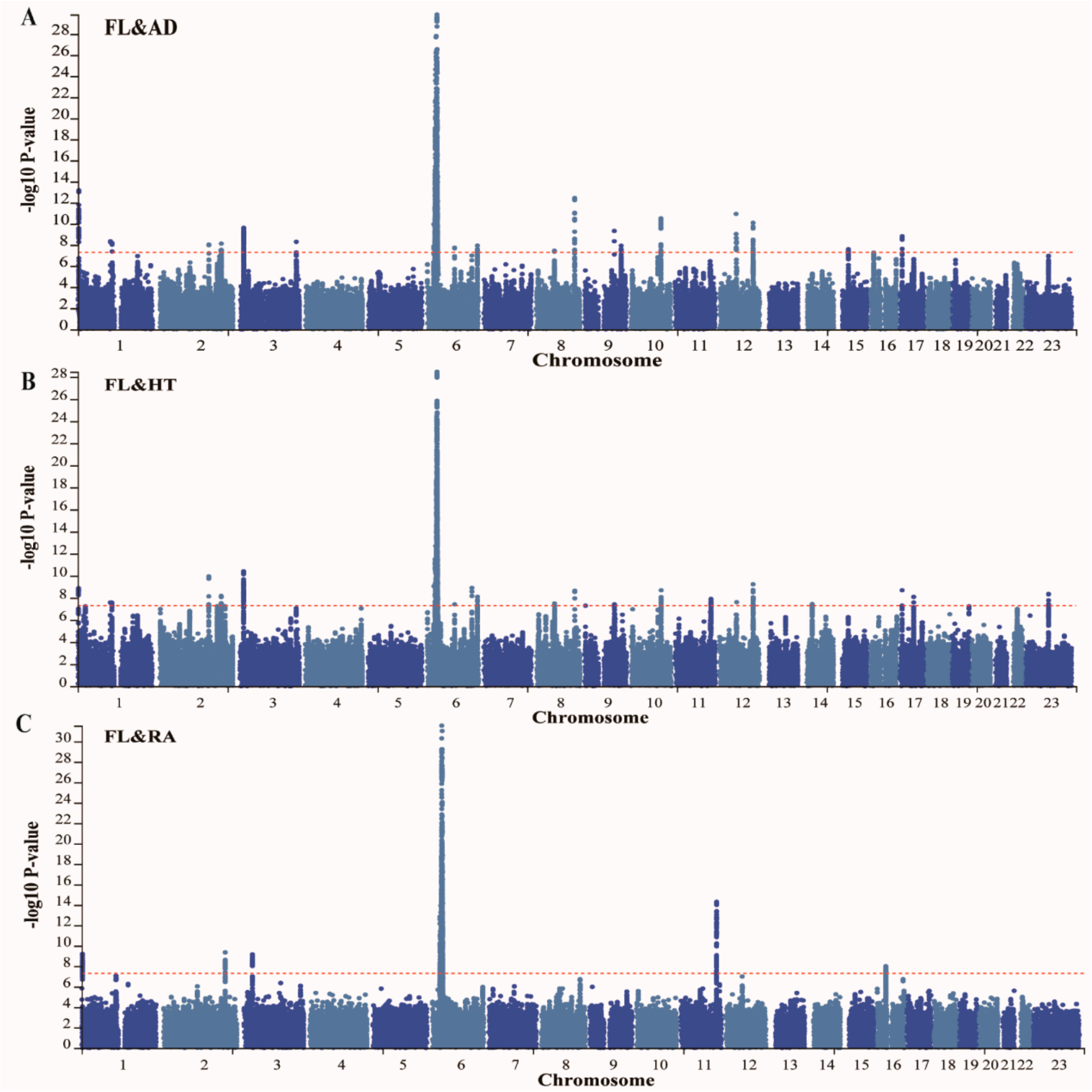

Figure S2. QQ plots for pleiotropic results performed by PLACO. Abbreviations: FL, Follicular lymphoma; AD, Composite autoimmune disease; RA, Rheumatoid arthritis; HT, Hypothyroidism.

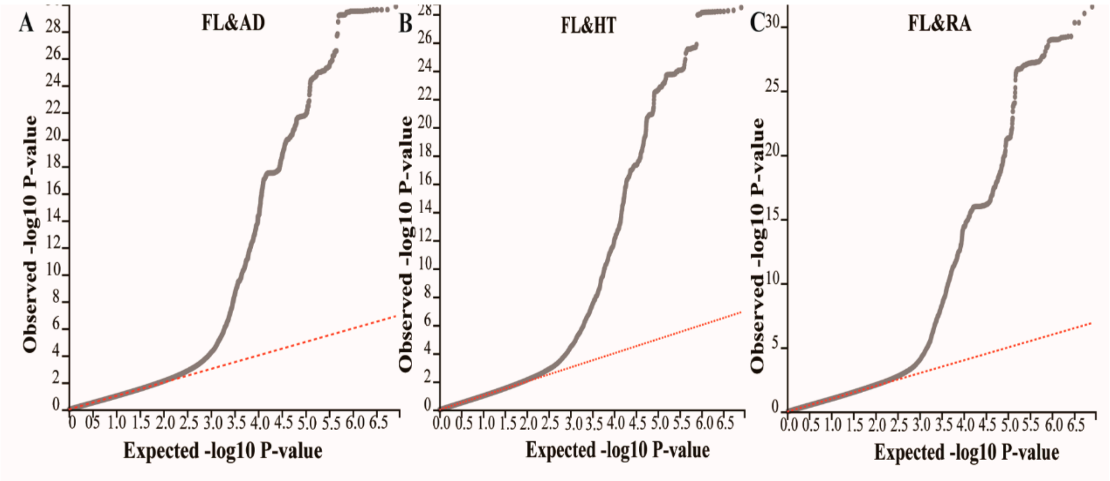

Figure S3. Key shared genetic loci between FL and AD. Note: Regional association plots for three genomic loci (A) 1p36.32, (B) 2q33.2, and (C) 6p21.32 that exhibited recurrent colocalization signals between FL and AD, HT, and RA. Although the PP.H4 for any single trait pair did not exceed the conventional threshold of 0.7, the recurrence of the same locus across multiple related trait pairs suggests these are key shared genetic regions. SNPs in LD that do not have any significant independent lead SNPs in the selected region are grayed out. For genes, mapped genes drawn by position mapping are in red; blue are unmapped protein-coding genes; dark gray are unmapped non-coding genes. Abbreviations: FL, Follicular lymphoma; AD, Composite autoimmune disease; RA, Rheumatoid arthritis; HT, Hypothyroidism; LD, linkage disequilibrium.

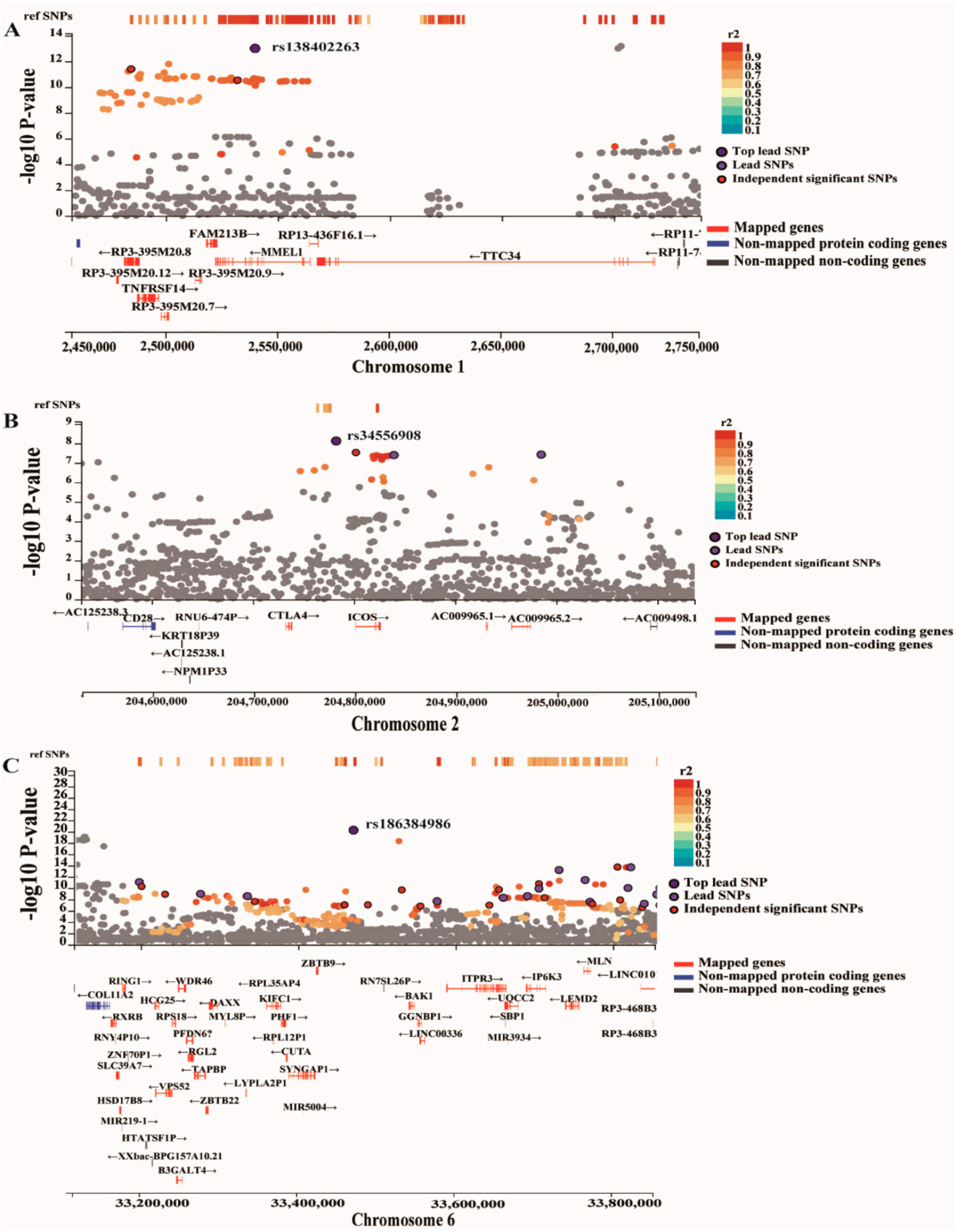

Figure S4. Key shared genetic loci between FL and RA. Note: Regional association plots for three genomic loci (A) 1p36.32, (B) 2q33.2, and (C) 6p21.32 that exhibited recurrent colocalization signals between FL and AD, HT, and RA. Although the PP.H4 for any single trait pair did not exceed the conventional threshold of 0.7, the recurrence of the same locus across multiple related trait pairs suggests these are key shared genetic regions. SNPs in LD that do not have any significant independent lead SNPs in the selected region are grayed out. For genes, mapped genes drawn by position mapping are in red; blue are unmapped protein-coding genes; dark gray are unmapped non-coding genes. Abbreviations: FL, Follicular lymphoma; AD, Composite autoimmune disease; RA, Rheumatoid arthritis; HT, Hypothyroidism; LD, linkage disequilibrium.

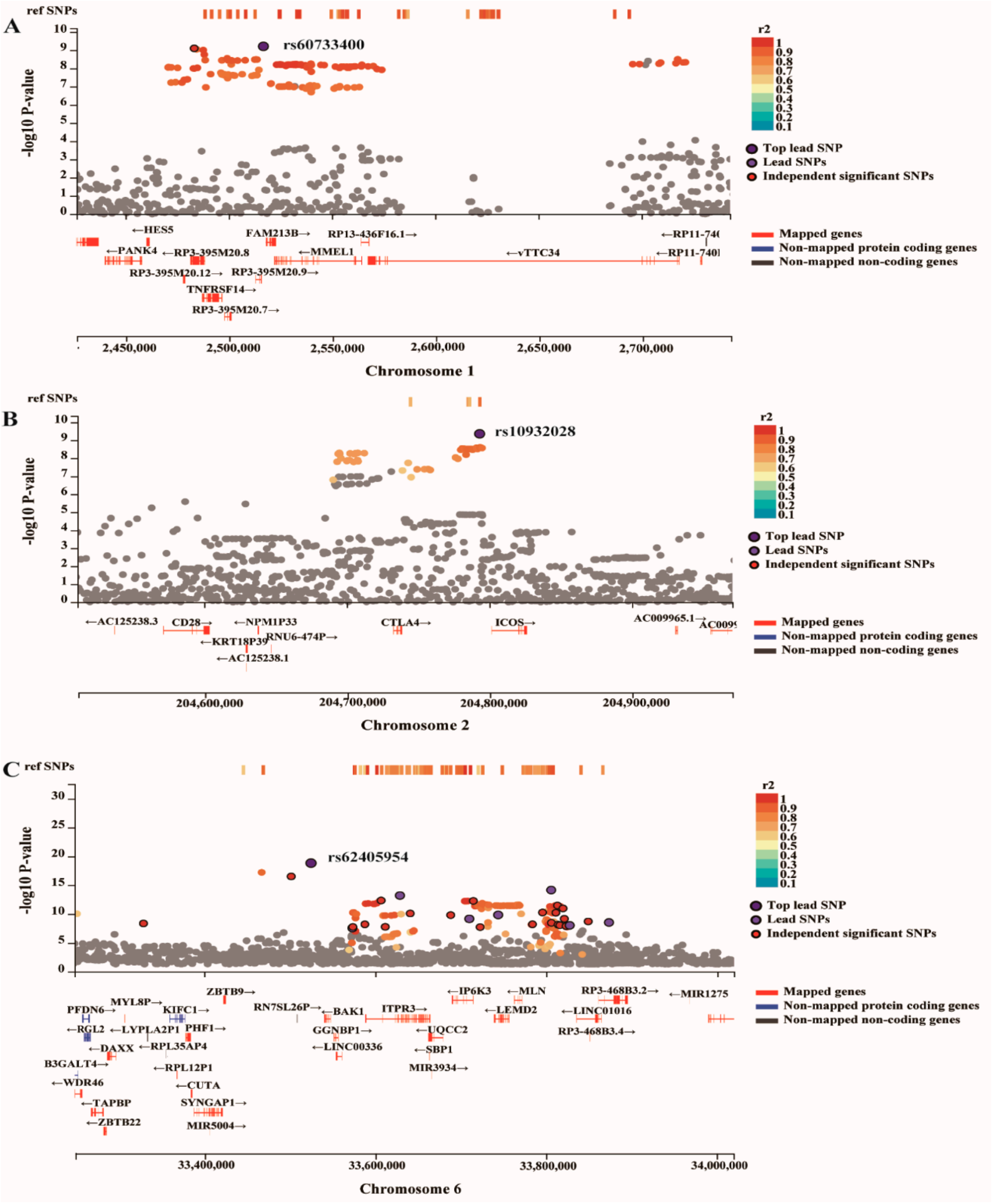

Figure S5. Key shared genetic loci between FL and HT. Note: Regional association plots for three genomic loci (A) 1p36.32, (B) 2q33.2, and (C) 6p21.32 that exhibited recurrent colocalization signals between FL and AD, HT, and RA. Although the PP.H4 for any single trait pair did not exceed the conventional threshold of 0.7, the recurrence of the same locus across multiple related trait pairs suggests these are key shared genetic regions. SNPs in LD that do not have any significant independent lead SNPs in the selected region are grayed out. For genes, mapped genes drawn by position mapping are in red; blue are unmapped protein-coding genes; dark gray are unmapped non-coding genes. Abbreviations: FL, Follicular lymphoma; AD, Composite autoimmune disease; RA, Rheumatoid arthritis; HT, Hypothyroidism; LD, linkage disequilibrium.

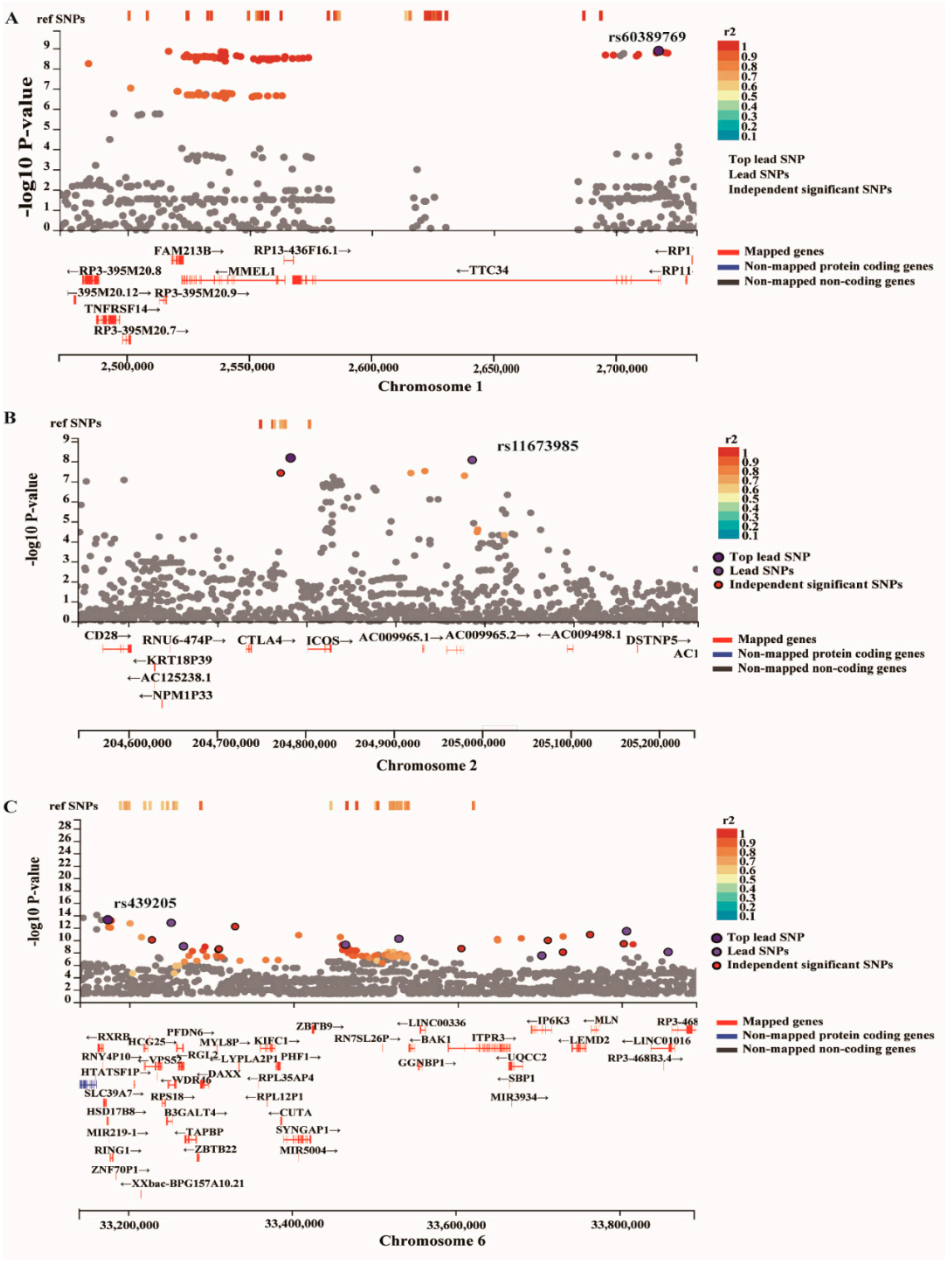

Figure S6. Regional association plots for loci with significant genetic colocalization between FL and AIDs. Regional plots of colocalized loci with PP.H4 ≥ 0.7 identified through PLACO analysis for FL with RA and AD. Note: (A-C) Loci 3p24.1, 11q23.3, and 16p12.1 represent significant shared genetic regions between FL and RA. (D) Locus 17p13.1 shows significant colocalization between FL and AD. SNPs in without independent significant associations are grayed out. Gene annotations: red indicates position-mapped genes; blue represents unmapped protein-coding genes; dark gray denotes unmapped non-coding genes. Abbreviations: FL, follicular lymphoma; AD, composite autoimmune disease; RA, rheumatoid arthritis; HT, hypothyroidism; LD, linkage disequilibrium.

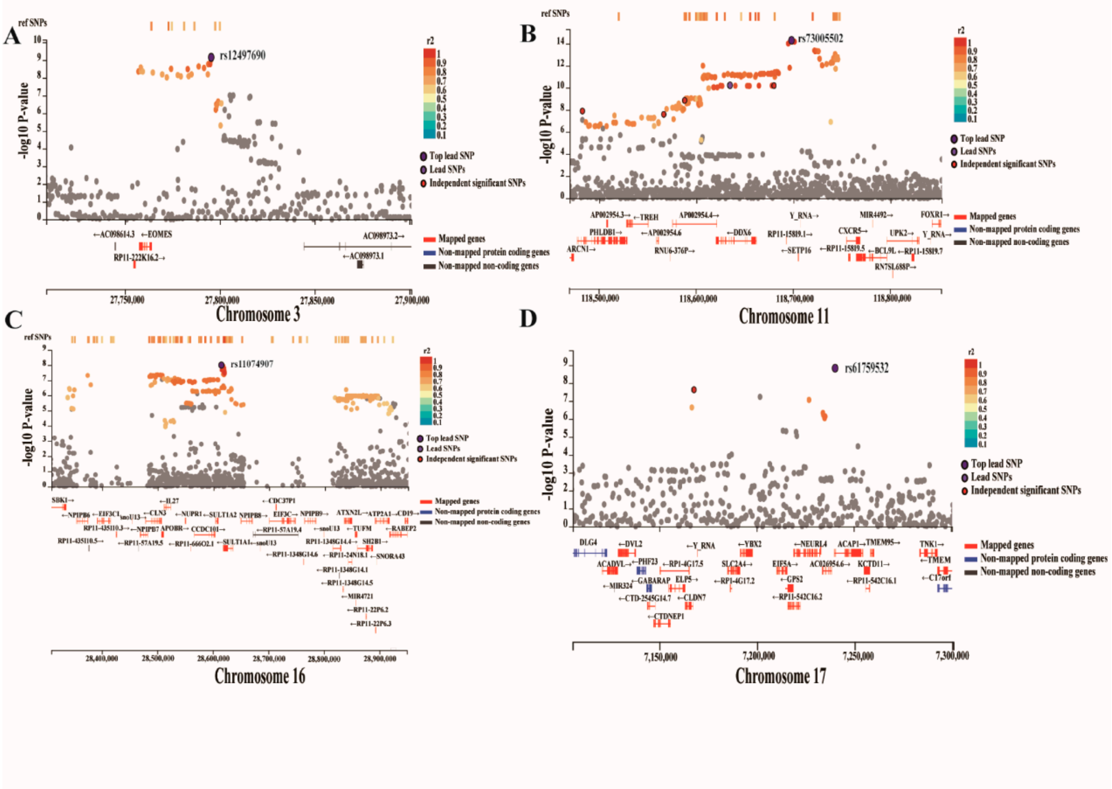

Figure S7. Tissue-specific expression patterns of pleiotropic genes. Note: Heatmap showing the expression levels (log2-transformed FPKM) of pleiotropic genes, identified by FUMA analysis, across a range of normal human tissues. Expression values are color-coded, with red representing high expression and blue representing low expression. Key tissues of interest for this study—whole blood, spleen, and EBV-transformed lymphocytes—are highlighted with black boxes, reflecting their relevance to immune function and lymphomagenesis.

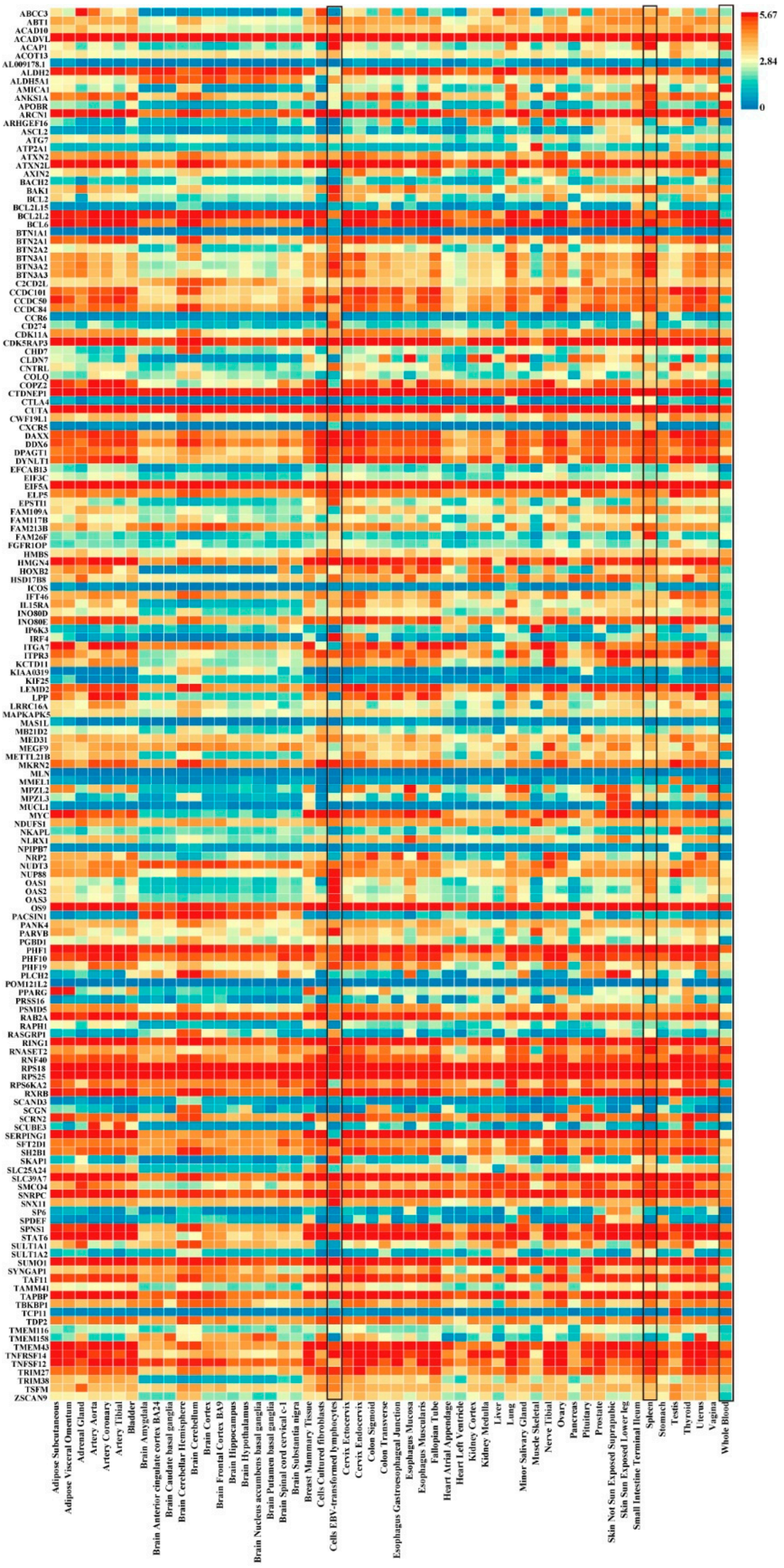

Figure S8. Gene-set enrichment for identified pleiotropic genes. Note: Red panels represent significant tissues after Bonferroni adjustment. Abbreviations: DEG, differentially expressed genes.

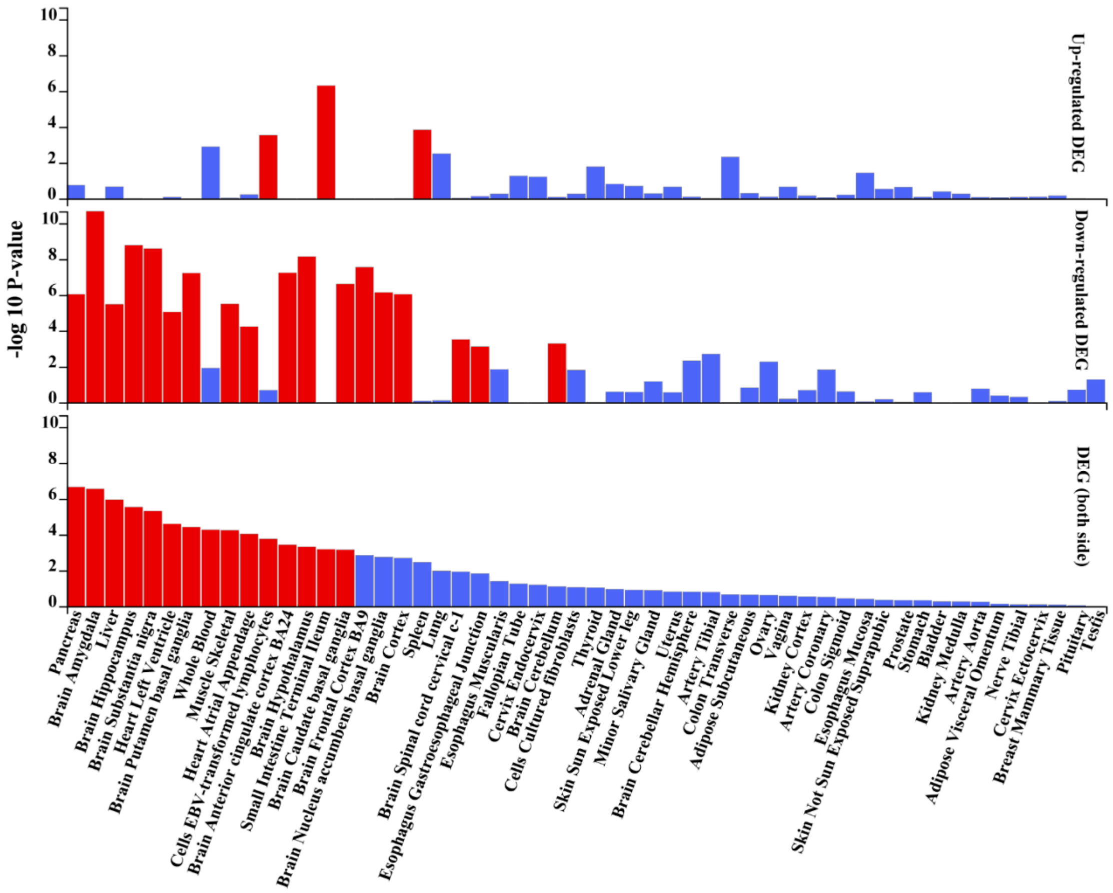

Supplement: Supplementary file 1 [file genes-17-01031-s001.zip › Supplementary Figures S1-S8.pdf]
